# Gait Complexity and Regularity Are Differently Modulated by Treadmill Walking in Parkinson's Disease and Healthy Population

**DOI:** 10.3389/fphys.2018.00068

**Published:** 2018-02-06

**Authors:** Thibault Warlop, Christine Detrembleur, Gaëtan Stoquart, Thierry Lejeune, Anne Jeanjean

**Affiliations:** ^1^Physical and Rehabilitation Medicine Department, Cliniques Universitaires Saint-Luc, Brussels, Belgium; ^2^Neuro Musculo Skeletal Lab, Institut de Recherche Expérimentale et Clinique, Université Catholique de Louvain, Brussels, Belgium; ^3^Louvain Bionics, Université Catholique de Louvain, Brussels, Belgium; ^4^Clinical Neuroscience (NEUR), Institute of Neurosciences (IoNS), Université Catholique de Louvain, Brussels, Belgium; ^5^Department of Neurology, Université Catholique de Louvain, Cliniques Universitaires Saint-Luc, Brussels, Belgium

**Keywords:** Parkinson's disease, gait variability, treadmill, gait analysis, locomotion, rehabilitation, nonlinear dynamics, fractals

## Abstract

Variability raises considerable interest as a promising and sensitive marker of dysfunction in physiology, in particular in neurosciences. Both internally (e.g., pathology) and/or externally (e.g., environment) generated perturbations and the neuro-mechanical responses to them contribute to the fluctuating dynamics of locomotion. Defective internal gait control in Parkinson's disease (PD), resulting in typical timing gait disorders, is characterized by the breakdown of the temporal organization of stride duration variability. Influence of external cue on gait pattern could be detrimental or advantageous depending on situations (healthy or pathological gait pattern, respectively). As well as being an interesting rehabilitative approach in PD, treadmills are usually implemented in laboratory settings to perform instrumented gait analysis including gait variability assessment. However, possibly acting as an external pacemaker, treadmill could modulate the temporal organization of gait variability of PD patients which could invalidate any gait variability assessment. This study aimed to investigate the immediate influence of treadmill walking (TW) on the temporal organization of stride duration variability in PD and healthy population. Here, we analyzed the gait pattern of 20 PD patients and 15 healthy age-matched subjects walking on overground and on a motorized-treadmill (randomized order) at a self-selected speed. The temporal organization and regularity of time series of walking were assessed on 512 consecutive strides and assessed by the application of non-linear mathematical methods (i.e., the detrended fluctuation analysis and power spectral density; and sample entropy, for the temporal organization and regularity of gait variability, respectively). A more temporally organized and regular gait pattern seems to emerge from TW in PD while no influence was observed on healthy gait pattern. Treadmill could afford the necessary framework to regulate gait rhythmicity in PD. Overall, the results support the hypothesis of a greater dependence to regulatory inputs as an explanatory factor of treadmill influence observed in PD. Also, since treadmill misrepresents the gait as more healthy than it is, the present findings underline that gait analysis using treadmill devices should be cautiously considered in PD and especially for gait variability assessment in gait lab.

## Introduction

Human movement is intrinsically dynamic and complex. Continuous integration of multiple sensory inputs and coordination of motor outputs are needed to achieve efficient, stable, and adaptable locomotion (Dingwell et al., 2010). Coordination among the multiple components and at multiple levels within the system reveals the complexity of physiological signals (Delignières and Marmelat, 2012). As such, human locomotion emerges from a wide set of interdependent elementary components, self-organized in coherent units and not dictated by a specific component within the system (Stergiou and Decker, 2011; Delignières and Marmelat, 2012).

Beyond this theoretical approach, complexity and adaptability appears tightly linked. Archetypally linked to the defective activity among interacting subcomponents (e.g., basal ganglia), the randomness of gait pattern observed in neurodegenerative diseases such as Parkinson's or Huntington's disease compromises adaptive gait control (Hausdorff et al., 1997; Warlop et al., 2016). Both externally (e.g., environment) and/or internally (e.g., pathology) generated perturbations and the neuro-mechanical responses to them contribute to the fluctuating dynamics of gait (Bohnsack-McLagan et al., 2016). Adaptability stems from a wide range of potential behaviors that is allowed by adopting coordinated fluctuating regimen (Stergiou and Decker, 2011).

Deviations from a structured optimal level of variability in either the direction of randomness (i.e., weak coordination level) or the over-regularity (i.e., strong coordination level) indicate the loss of the adaptive capabilities of the system (Stergiou and Decker, 2011). In human locomotion, perturbations of the temporal organization of gait variability have been suggested as a marker of gait disorder and fall risk among patients with central nervous diseases such as Parkinson's disease (PD) or Huntington's disease (Hausdorff et al., 1997, 2000; Warlop et al., 2016).

Interestingly, the use of assistive devices can modulate the temporal organization of gait variability in PD (Warlop et al., 2017). Beneficial effects of external sensory inputs from various natures (auditory, visual, tactile,…) were demonstrated to compensate or restore interactions among multiple components involved in healthy gait pattern (Hunt et al., 2014; Marmelat et al., 2014a,b; Chien et al., 2017). By providing the necessary trigger, external sensory cues allow to bypass the defective pallidocortical circuit in PD (van Wegen et al., 2006; Baker et al., 2007; Nieuwboer et al., 2007).

Treadmill training, recognized as a task-oriented approach for gait rehabilitation in the PD population, could act as an external pacemaker (Frenkel-Toledo et al., 2005; Bello and Fernandez-Del-Olmo, 2012). Improving stride length and gait speed, treadmill walking (TW) was suggested as a more optimal condition for the PD patients to generate a normal gait pattern compared to overground walking (Bello and Fernandez-Del-Olmo, 2012). In addition, as well as being an interesting rehabilitative approach in PD, treadmills are usually implemented in laboratory settings to perform instrumented gait analysis (Chang et al., 2009; Bollens et al., 2010). Although no treadmill effect was demonstrated in healthy population walking at a confortable speed and individually determined (Chang et al., 2009; Bollens et al., 2010), potential treadmill effect during gait assessment needs thus to be considered in the specific context of PD.

Despite the frequent use of treadmill in research and rehabilitation fields, the influence of such type of locomotion on gait variability was poorly investigated in the specific context of Parkinson's disease. Only one study has compared the temporal organization of gait variability on treadmill and overground walking in PD while the treadmill walking influence on gait variability was the main focus of numerous studies conducted on healthy adults (Frenkel-Toledo et al., 2005). In 2005, these authors suggested that TW could act as an external cue to enhance gait rhythmicity and reduce gait variability. However, it is noteworthy to mention that both the temporal organization and magnitude of gait variability (i.e., the coefficient of variation of gait variability) were assessed on walking session of 2 min, while both gait variability approaches are usually data-hungry techniques (Delignieres et al., 2006; Crevecoeur et al., 2010; Damouras et al., 2010). Gait variability measures should be respectively assessed on 512 and 127 consecutive gait cycles to draw adequate conclusions (Riva et al., 2014). Hence, the practical challenge of capturing long-time series in pathological population is not trivial. Furthermore, the TW influence on severely affected patients and the role of fear of falling remain to be determined.

Therefore, considering the widespread attention paid on treadmill in the last decade in both rehabilitation and gait analysis and the necessity to reliably assess gait variability, we investigate whether TW influences the fluctuation dynamics of locomotion in PD. Given that treadmill ranks among rehabilitation approaches in PD (Mehrholz et al., 2015), we put forward the hypothesis that the temporal organization of variability would be more structured by external inputs from treadmill. On the contrary, the magnitude would be more pronounced on the moving surface of treadmill, potentially reflecting fear of falling. Since the gait automaticity and balance performance tend to decrease with the disease progression, the third hypothesis is that patient severely affected could benefit the most from TW.

## Materials and methods

### Subjects

Twenty participants suffering from idiopathic PD, diagnosed according to the UK Brain Bank Criteria (Hughes et al., 1992), and 15 healthy age-matched controls were recruited from the local community and local Parkinson's disease Association. A mandatory requirement for inclusion in the study was the ability to perform 10 min of walking without walking aids or assistance. PD patients were excluded if they had a past history of any neurological condition other than PD, orthopedic, or visual disturbance that affected walking ability. The study was approved by the local ethics committee (Comité d'Ethique Hospitalo-Facultaire de l'Université catholique de Louvain). Prior to data collection, all participants were informed about the experimental protocol and gave written consent in accordance with the Declaration of Helsinki.

### Procedure

Prior to walking tests, each participant underwent a complete functional assessment including the subjects' performance on a balance test (BESTest) and the fear of falling (ABC-Scale). For PD participants, the disease severity (modified Hoehn & Yahr scale) and the patient's functional status (MDS-UPDRS) were also determined. All assessments were carried out during the ON state.

Each participant performed two walking sessions of 10 min for obtaining 512 consecutive strides that is required to adequately apply mathematical methods described below (Crevecoeur et al., 2010). Both sessions consisted of walking at a self-selected speed around a 42 m oval indoor track (overground walking-OW) and on a motor-driven treadmill (treadmill walking (TW); Mercury LT med, HP Cosmos, Germany). All participants were naïve to the treadmill walking. In order to avoid fatigue and any gait rehabilitation benefits, the two walking conditions were spread over 2 days and the order of sessions was randomly allocated. With respect to the treadmill walking session, each patient was instructed to walk on the treadmill at a comfortable walking speed without holding the handrails. The treadmill speed was adjusted to match each participant's comfortable speed (Bayat et al., 2005; Chien et al., 2017). This comfortable speed was determined following procedures used by Bayat et al. with patients suffering from stroke and Chien et al. with elderly individuals (Bayat et al., 2005). A gradual increase by increments of 0.10 m/s, initiated at 0.15 m/s, was executed until the subject indicated that the speed was too fast. In that case, the speed was decreased by 0.10 m/s, determining the comfortable gait speed. Once the patient reached his own comfortable speed, a familiarization period of 5 min was respected. Participants were then asked to perform the walking session of 10 min. In order to ensure patient security, a safety harness was used (LiteGait®, Mobility Research, Inc.). However, the system was adjusted not to avoid to support the subject (0% body weight support) in order to not influence patient's stride time dynamics (Stout et al., 2016).

Immediately after the specific familiarization period (i.e., one lap for overground walking session and 5 min for treadmill walking session), data acquisition started and the 512 first gait cycles were extracted for further analysis. In each of the two walking conditions, a unidimensional accelerometer was taped in the antero-posterior direction on the lateral malleolus of the most affected side in PD participants and the dominant side in healthy controls. Acceleration data were recorded at 512 Hz using the Vitaport 3 ambulatory recorder (Temec Instruments B.V., Kerkrade, The Netherlands), and next transferred to a computer. Heel strike was detected in the raw acceleration signal with a peak detection method designed to minimize the risk of false step detection (Terrier and Dériaz, 2011; Fortune et al., 2014). Validated against ground reaction forces, this method assumes that each acceleration peak corresponds to successive foot contact (Sinclair et al., 2013). The stride duration was determined from time elapsed between successive acceleration peaks, easily and accurately revealed by the use of a high sampling rate.

The stride duration variability was the primary outcome of our study and was investigated both in terms of magnitude, using standard metrics such as sample mean, standard deviation (SD) and coefficient of variation (CV), and in terms of its organization, which provides complementary information about how stride duration evolves with time across consecutive strides (Stergiou and Decker, 2011; Bollens et al., 2014; Warlop et al., 2016). In human locomotion, stride duration fluctuates in a structured, complex manner over the long term, displaying the presence of long-range autocorrelations (LRA) that can span hundreds of consecutive strides. For each time series, the magnitude and the temporal organization of stride duration variability were applied to sequences of 512 consecutive gait strides. The presence of LRA was evaluated, using an integrated approach that combines the results of distinct non-linear mathematical methods. The Detrended Fluctuation Analysis (DFA) and the Power Spectral Analysis (PSD) were applied to compute the Hurst exponent and the α exponent, respectively. Those methods were preferred given their relevance on both stationary and non-stationary processes (Delignieres et al., 2006).

The Detrended Fluctuation Analysis (DFA) requires to initially integrate the x(*t*) series by computing for each *t* the accumulated departure from the mean of the whole series (Delignieres et al., 2006):

$$\begin{array}{l} X\left(k\right)=\overset{k}{\underset{i=1}{\sum }}\left[x\left(i\right)-\bar{x}\right] \end{array}$$

This integrated series is divided into non-overlapping intervals of length *n*. In each interval, a least squares line is fit to the data (representing the trend in the interval). The series X(*t*) is then locally detrended by subtracting the theoretical values X_n_(*t*) given by the regression. For a given interval length *n*, the characteristic size of fluctuation for this integrated and detrended series is calculated by Delignieres et al. (2006):

$$\begin{array}{l} F=\sqrt{\frac{1}{N}}\overset{N}{\underset{k=1}{\sum }}{\left[X\left(k\right)-X_{n}\left(k\right)\right]}^{2} \end{array}$$

This procedure is applied to subseries of increasing size from *n* = 10 to *n* = *N*/2 and averaged across subsets of equal size. The slope of the relationship between log (F) and log (*n*) determined H exponent.

The Power Spectral Density (PSD) of the time series was calculated with Fourier transform analysis, and α exponent was estimated from the slope of a linear regression on a log (power) vs. log (frequency) plot (Bollens et al., 2010).

To increase the level of confidence in the results, the consistency of H and α exponents was verify through the asymptotic relationship: $d=H-\;\left[\frac{\left(1+\alpha \right)}{2}\right].$.

Following such integrated approach, the three following criteria must be met to conclude the presence of LRA:

- 0.5 < *H* ≤ 1
- α significantly different from 0 and lower than 1
- *d* ≤ 0.10

Further analyses were applied when *H* and α violate their asymptotic relationship under the hypothesis of a long-range auto-correlated process. In the present study, we used the randomly shuffled surrogate data test, described by Theiler et al. (1992). For each time series, 100 new series are generated from a random permutation of the original data. The only difference between the surrogate data sets and the original series is the sequential ordering of the data. *H* and α are then calculated for each new series and the mean and SD of these 100 new scaling exponents are computed and compared with the exponents of the original time series. The number of standard deviation between the original scaling exponent and the mean scaling exponent of the surrogate data sets (σ) is computed. If σ > 2, then the difference between the original data set and the surrogate data set is considered statistically different. It does not prove the presence of long-range autocorrelations in the original time series but it can reject the null hypothesis that the series under investigation has no temporal structure (i.e., uncorrelated random process).

Sample Entropy (SampEn) was also calculated on 512 consecutive strides to assess the regularity or predictability of the lower-limb movement. Smaller value is associated with the greater regularity whereas a system with high SampEn appears less predictable; generating more non-redundant information over time. Since SampEn values were influenced depending on input parameter, *m* (i.e., length of the data segment being compared) and *r* (similarity criterion) were set according to the recommendations of Yentes et al. Values of 2 and 0.2 were respectively used for *m* and *r* (Yentes et al., 2013).

For further details, these methods are described elsewhere (Theiler et al., 1992; Rangarajan and Ding, 2000; Delignieres et al., 2006; Crevecoeur et al., 2010; Yentes et al., 2013).

Classical spatio-temporal gait parameters, assessed during both overground and treadmill walking sessions, constitute the secondary outcomes. During the treadmill walking session, the mean gait speed is known and the gait cadence is extracted from the accelerometer data. By identifying heel strikes (peak detection method), the total number of steps can be determined from the raw acceleration signals. The step length was assessed following the relationship between the gait speed and gait cadence. During overground walking, lap times, laps number and the total distance were measured. Following the relationship between total walking distance and acquisition duration, mean gait speed, mean gait cadence and mean step length were assessed as follow:

$$\begin{array}{lll} Gait\;speed\;(m.s^{-1}) & = & \frac{Total\;walking\;distance\;\left(m\right)}{Acquisition\;duration\;\left(s\right)};\\ Gait\;cadence\;(\#steps.min^{-1}) & = & \frac{Total\;number\;of\;steps\;\left(\#\right)}{Acquisition\;duration\;\left(min\right)};and\\ Step\;length\;\left(m\right) & = & \frac{Gait\;speed}{Gait\;cadence}. \end{array}$$

### Statistical analysis

Data were analyzed with the Sigmaplot 13.0 software (Systat, Richmond, CA, USA). Standard measures of statistical dispersion [mean (±standard deviation) and median (interquartile range) for normally and non-normally distributed data sets, respectively] were computed to describe the study populations. Normality of data distribution was verified (using the Shapiro-Wilk test) for all variables, except for the gait cadence and the CV of stride duration that required the use of a log-transformation. A two-way repeated measures ANOVA (Walking condition × Pathological condition) was applied to examine the influence of walking condition, as well as one of the pathological condition on LRA exponents, CV of stride duration and spatiotemporal gait variables. The effect size of TW as compared to OW was expressed in standardized terms (Hedge's g). Most of gait variables are speed- and/or age-dependent. Thus, to further identify the walking condition-caused gait modulations as opposed to changes arising solely from the age of subjects or from different walking speeds, all gait variables were age- and speed-normalized using a Z-score transformation. Andriacchi et al. and Winter's reference works provided normative data of spatiotemporal gait variables (Andriacchi et al., 1977; Winter, 1991). Given that no normative values for LRA exponents are currently established and that no influence of age and gait speed were demonstrated, values from our healthy control population were used as a reference point. Whereas the influence of gait speed on the CV of stride duration was highlighted at low speeds (i.e., 0.2–0.6 m.s-1), most of our PD participants walked at a gait speed above 0.6 m.s-1. Thus, considering this aspect and the absence of normative data for the CV of stride duration, healthy control values were also used as a reference point. Subjects Z-transformed gait variables on OW and TW were compared using a paired *t*-test. The treadmill influence in PD was also further investigated with respect to clinical factors such as disease severity, balance performance or fear of falling using Spearman's correlation coefficients. The results were considered statistically different for *p* < 0.05.

## Results

Anthropometric and clinical characteristics of the PD and healthy control groups are summarized in Table 1. Both groups were similar with respect to age, gender, height, weight. Significantly lower scores were reached in balance performance, balance confidence and cognitive function among PD participants in comparison with the healthy age- and gender-matched controls. Three PD patients suffered from motor complications in their daily-life (one from freezing of gait and two from dyskinesia (trunk or upper limb). However, none of such complications hindered the two walking acquisitions of those patients.

**Table 1:** Characteristics of the study populations.

	**Parkinson's disease (*n* = 20)**	**Healthy (*n* = 15)**	***p*-value**
Age (years)	65.3 ± 9.6	60.1 ± 13.3	0.166
Gender	11 (male) and 9 (female)	9 (male) and 6 (female)	
Height (cm)	170.3 ± 9.7	171.8 ± 5.4	0.600
Weight (kg)	74.0 ± 17.1	67.0 ± 10.0	0.168
Time since the diagnosis (years)	4.5 ± 2.7	–	
MMSE score	29.0 [24–30]	30.0 [29–30]	0.014
H&Y scale	2 [1–3]	–	
Most affected/dominant side	13 (left) and 7 (right)	2 (left) and 13 (right)	
MDS-UPDRS III (/132)	24.0 [9–66]	–	
MDS-UPDRS total (/260)	49.0 [15–133]	–	
BESTest total (%)	78.0 [49–92]	88.0 [82–90]	0.005
ABC Scale (%)	78.0 [54–100]	95.0 [76–98]	0.001

*Mean (± SD) are expressed for quantitative variables normally distributed while median [range] are expressed for both ordinal and non-normally distributed variables*.

### Treadmill effect on PD and healthy gait pattern

**Figure 1:**
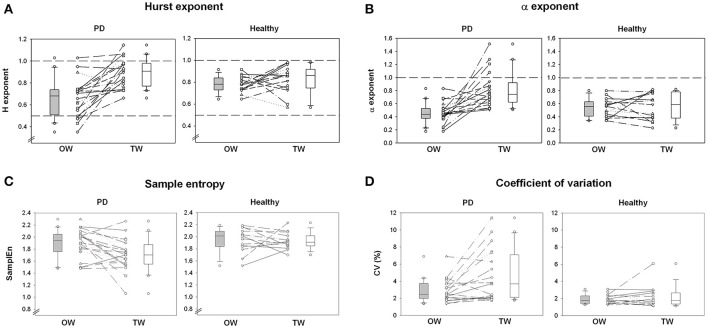
Stride duration variability. **(A)** Shows individual changes of Hurst exponent for overground walking (OW) and treadmill walking (TW) in PD and healthy populations. **(B)** Shows individual changes of α exponent between walking conditions and study populations. **(C)** Shows individual changes of sample entropy between walking conditions and study populations. **(D)** Shows individual changes of the coefficient of variation of the stride duration variability between walking conditions and study populations. Boxplots represent the median and the quartiles.

**Figure 2:**
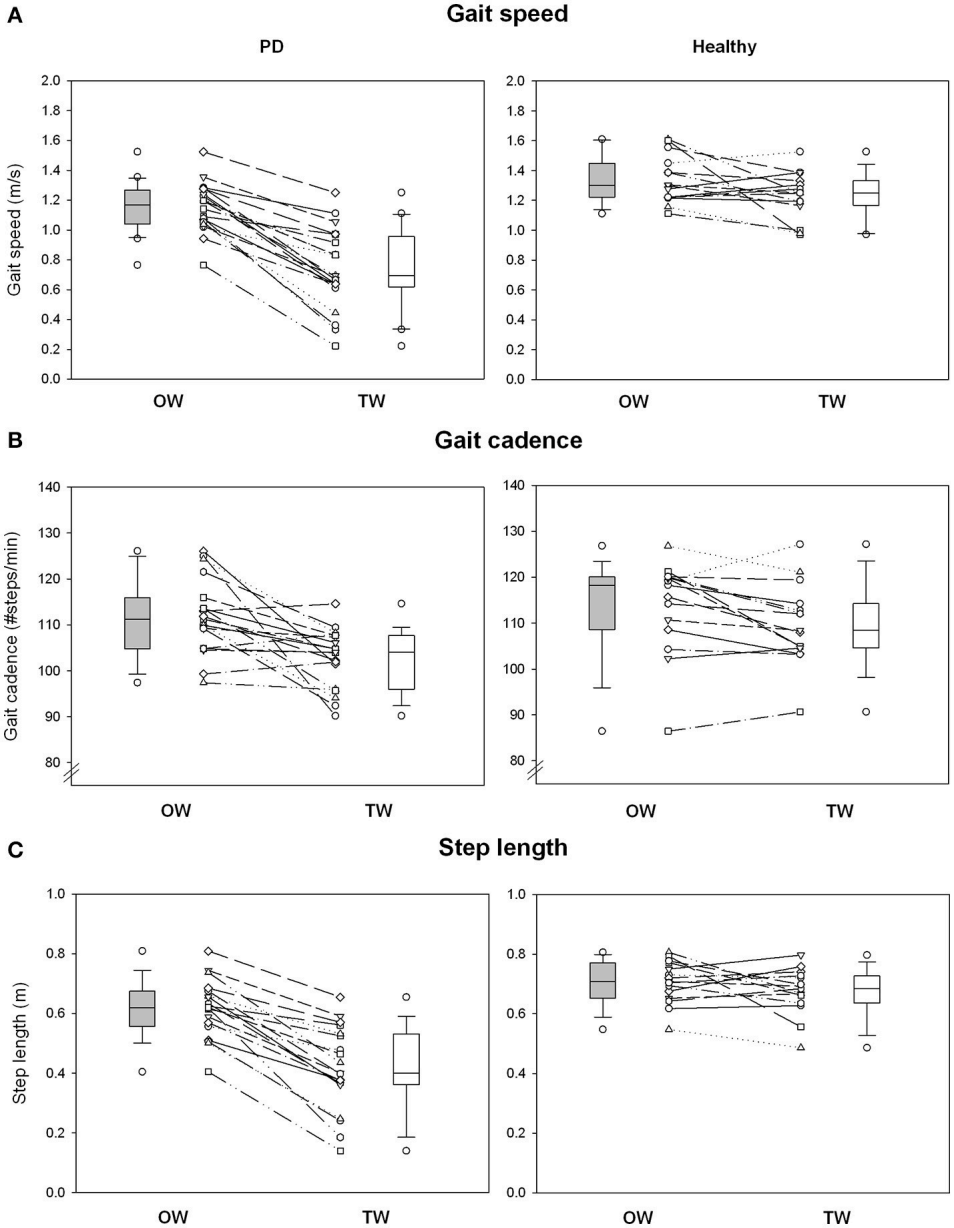
Spatiotemporal gait variables. **(A)** Shows individual changes of gait speed for overground walking (OW) and treadmill walking (TW) in PD and healthy populations. **(B)** Shows individual changes of gait cadence between walking conditions and study populations. **(C)** Shows individual changes of step length between walking conditions and study populations. Boxplots represent the median and the quartiles.

**Figure 3:**
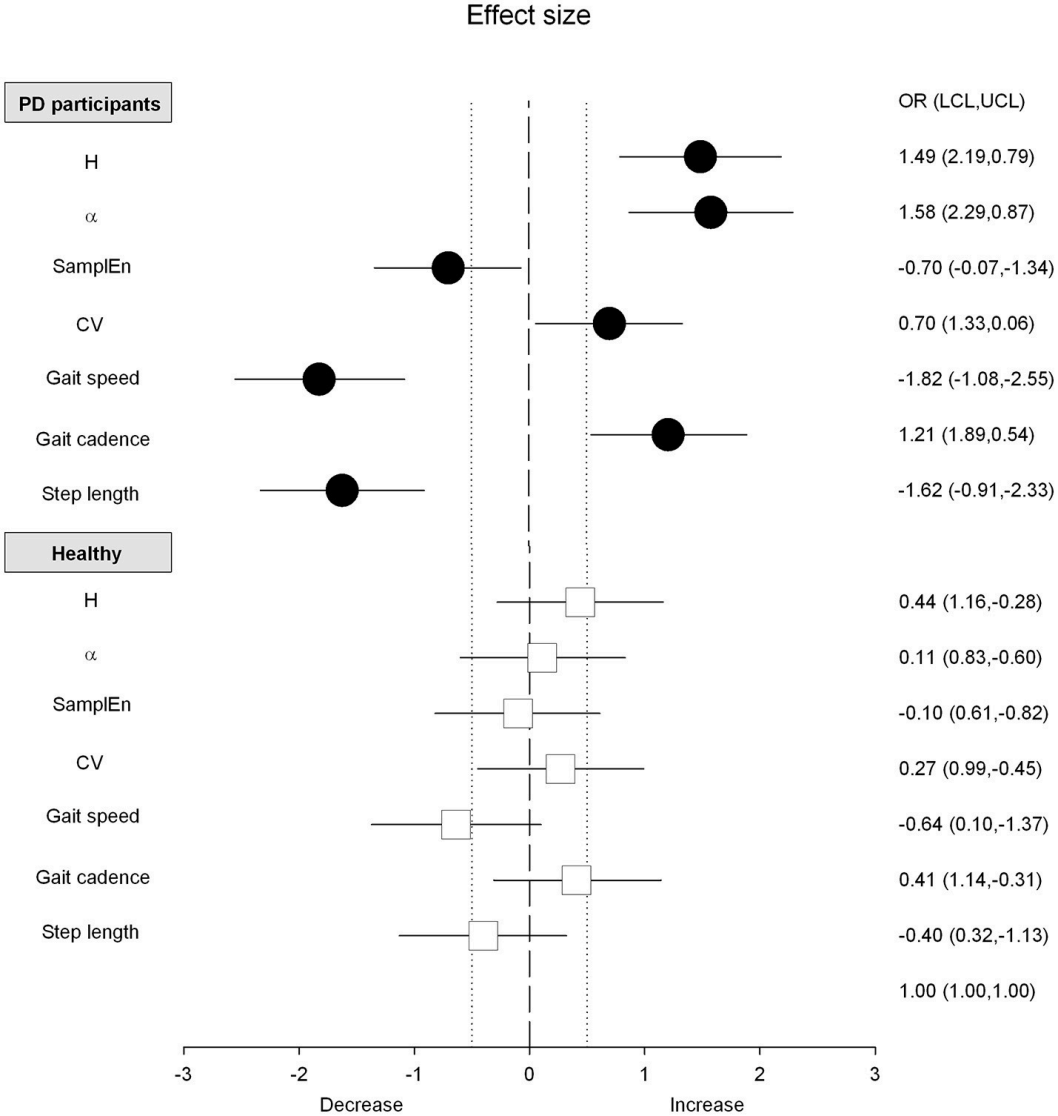
Effect size and confidence intervals for the differences between overground and treadmill walking. Black circles (PD patients) and white squares (Healthy controls) are the standardized effect size (Hedge's g). Horizontal lines are the 95% confidence intervals. Vertical dotted lines, arbitrarily fixed at 0.5 and−0.5, correspond to a medium effect as defined by Cohen.

Absolute and normalized values of stride duration variability measures and spatio-temporal gait variables are summarized in Figures 1, 2 and Tables 2, **4**. The standardized effect-size (Hedge's g) is depicted for the PD participants and healthy controls in Figure 3.

**Table 2:** Absolute mean values of the stride duration variability and spatiotemporal gait variables for the comparison between the overground walking (OW) and the treadmill walking (TW) sessions in Parkinson's disease and healthy subjects.

	**Parkinson's disease**				**Healthy controls**			
	**OW**	**TW**	***t***	***p*-value**	**OW**	**TW**	***t***	***p*-value**
**STRIDE DURATION VARIABILITY**								
H exponent	0.66 (±0.17)	0.89 (±0.14)	−4.925	**≤0.001**	0.78 (±0.07)	0.82 (±0.12)	−1.162	0.264
α exponent	0.46 (±0.16)	0.81 (±0.27)	−4.595	**≤0.001**	0.54 (±0.14)	0.56 (±0.20)	−0.376	0.713
Sample Entropy	1.89 (±0.24)	1.71 (±0.27)	2.688	**0.015**	1.94 (±0.19)	1.93 (±0.13)	0.302	0.767
CV (%)	2.88 (±1.34)	4.53 (±3.00)	−2.932	**0.009**	1.91 (±0.54)	2.18 (±1.25)	−0.912	0.377
**SPATIOTEMPORAL GAIT VARIABLES**								
Gait speed (m/s)	1.15 (±0.16)	0.74 (±0.27)	9.929	**≤0.001**	1.34 (±0.16)	1.23 (±0.16)	2.060	0.059
Gait cadence (#steps/min)	111.85 (±8.12)	102.78 (±6.46)	3.934	**≤0.001**	113.83 (±10.13)	109.80 (±8.83)	2.411	**0.030**
Step length (m)	0.61 (±0.09)	0.41 (±0.14)	9.147	**≤0.001**	0.70 (±0.07)	0.67 (±0.07)	1.438	0.173

*Absolute value is expressed as mean (± SD). Bold data indicates if the comparison between UW and NW (paired t-test) was significant (p < 0.05)*.

The PD gait pattern was greatly influenced by TW while measures of gait variability and classical spatio-temporal gait variables remained similar in healthy controls (Figures 1, 2 and Table 2). In PD participants, TW was on averaged performed at lower gait speed than on OW, associated with a decrease of both gait cadence and step length (Figure 2 and Table 2). In comparison to the more unpredictable and random gait pattern observed on overground in PD, a more regular and temporally organized (i.e., decrease of SampEn and increase of H and α exponent, respectively) gait pattern seemed to emerge from TW (Figure 1 and Table 2). While values of H lower than 0.5 were highlighted for five PD participants on OW, values of H and α exponents closer to 1 or even higher than 1 for some participants were reached on TW. Values of *d* were below 0.10 in healthy population and both walking conditions (0.06 ± 0.02 and 0.07 ± 0.08 for OW and TW, respectively) while it reached values higher than 0.10 in PD population (0.12 ± 0.10 and 0.11 ± 0.10 for OW and TW, respectively). Using the surrogate data tests, the presence of a specific temporal structure in original time series can still be suggested for time series that did not verify the H, α, and/or *d* criteria. Across all conditions, the number of standard deviations between the original scaling exponent and the scaling exponent of the surrogate series (σ_*H*_ and σ_α_) were on average clearly superior to 2 for both populations. Higher magnitude of stride duration variability (i.e., increase of CV stride duration) was also induced by the TW. Importantly, significant (Walking condition × Pathological condition) interactions were demonstrated for LRA exponents, and the gait speed and step length confirming that TW influenced differently the gait pattern of PD and healthy participants (Table 3).

**Table 3:** Results of a Two-Way Repeated Measures ANOVA with walking condition (OW and TW) and pathological condition (Healthy controls and PD) as main factors on stride duration variability and spatiotemporal gait variables.

					**Two-Way repeated measures ANOVA**					
	**Parkinson's disease**		**Healthy controls**		**Walking condition**		**Pathological condition**		**Walking** × **Pathological**	
	**OW**	**TW**	**OW**	**TW**	***F***	***p*-value**	***F***	***p*-value**	***F***	***p*-value**
**STRIDE DURATION VARIABILITY**										
H exponent	0.66 (±0.17)	0.89 (±0.14)	0.78 (±0.07)	0.82 (±0.12)	26.383	**<0.001**	0.999	0.325	12.193	0.088
α exponent	0.46 (±0.16)	0.81 (±0.27)	0.54 (±0.14)	0.56 (±0.20)	13.772	**<0.001**	3.727	0.062	10.921	**0.001**
Sample Entropy	1.89 (±0.24)	1.71 (±0.27)	1.94 (±0.19)	1.93 (±0.13)	4.572	**0.040**	5.555	**0.025**	3.088	**0.002**
CV (%)	2.88 (±1.34)	4.53 (±3.00)	1.91 (±0.54)	2.18 (±1.25)	6.934	**0.013**	12.306	**0.001**	3.698	0.063
**SPATIOTEMPORAL GAIT VARIABLES**										
Gait speed (m/s)	1.15 (±0.16)	0.74 (±0.27)	1.34 (±0.16)	1.23 (±0.16)	63.757	**<0.001**	32.637	**<0.001**	23.055	**<0.001**
Gait cadence (#steps/min)	111.85 (±8.12)	102.78 (±6.46)	113.83 (±10.13)	109.80 (±8.83)	18.634	**<0.001**	3.067	0.089	3.160	0.085
Step length (m)	0.61 (±0.09)	0.41 (±0.14)	0.70 (±0.07)	0.67 (±0.07)	49.672	**<0.001**	41.188	**<0.001**	29.263	**<0.001**

*Bold data indicates statistically significant differences (p < 0.05)*.

As PD walked at their own comfortable speed that differed on OW and TW, normalized values (Z-score) were used to refine previous observations and draw adequate conclusions by taking into account gait speed differences observed between the two walking sessions (Table 4). Interestingly, the statistical difference based on absolute values highlighted for the CV stride duration became non-significant using normalized values while all other comparisons led to the same conclusions (Table 4). Adequate comparisons with healthy controls were also allowed by the use of normalized values. In comparison to the healthy population, the reduced gait-speed and step length and the increased CV highlighted on OW were intensified on TW. On the contrary, the random gait pattern observed on OW tended to be performed at a higher gait cadence and tended to be more regular and organized on TW than healthy controls.

**Table 4:** Mean values of the normalized stride duration variability and spatiotemporal gait variables (Z score) for the comparison between the overground walking (OW) and the treadmill walking (TW) sessions in Parkinson's disease.

	**Z-score*****-*****Paired** ***t-*****test**			
	**OW**	**TW**	***t***	***p*-value**
**STRIDE DURATION VARIABILITY**				
H exponent	−1.71 (±2.48)	0.46 (±1.02)	−4.934	**≤0.001**
α exponent	−0.58 (±1.13)	1.33 (±1.35)	−4.319	**≤0.001**
Sample Entropy	−0.38 (±1.26)	−1.60 (±1.90)	2.911	**0.009**
CV (%)	1.81 (±2.49)	1.88 (±2.39)	−0.143	0.888
**SPATIOTEMPORAL GAIT VARIABLES**				
Gait speed (m/s)	−1.72 (±1.13)	−4.71 (±1.95)	9.929	**≤0.001**
Gait cadence (#steps/min)	−0.19 (±1.42)	1.15 (±1.84)	−5.314	**≤0.001**
Step length (m)	−0.53 (±0.52)	−1.05 (±0.42)	5.739	**≤0.001**

*Z-score is expressed as mean (± SD). Bold data indicates if the comparison is significantly different (p < 0.05)*.

### Clinical status influence on walking behavior

As depicted on Figures 1, 2, some PD participants tended to be highly influenced by the treadmill while no influence was observed for others. To further investigate potential factors that could explain such distinct behaviors, Spearman's correlation coefficients were calculated between gait changes induced by the treadmill and the patients' clinical status. Table 5 reported all Spearman's correlation coefficients and its corresponding *p*-values. The temporal organization appeared highly related to the patient's functional status, and in particular its balance performance, while the magnitude of the stride duration variability was related to the gait speed changes (*r* = −0.51, *p* = 0.022). H and α exponents were respectively moderately and highly correlated to the disease severity (H&Y; *r* = 0.60, *p* = 0.004 for H and *r* = 0.75, *p* ≤ 0.001 for α, respectively). MDS-UPDRS III and BESTest were moderately correlated to H and α exponents (MDS-UPDRS III; *r* = 0.56, *p* = 0.013 for H and *r* = 0.58, *p* = 0.008 for α, respectively; BESTest; *r* = −0.51, *p* = 0.021 for H and *r* = −0.64, *p* = 0.002 for α, respectively). Similarly, the step length was correlated to both balance performance and confidence (BESTest; *r* = 0.73, *p* ≤ 0.001 and ABC-Scale; *r* = 0.66, *p* = 0.002). The balance status could be thus the explanatory factor of the important changes observed between the two walking sessions in some patients.

**Table 5:** Correlations study between the changes of both stride duration variability and gait parameters with clinical parameters in Parkinson's disease.

	**Changes between overground and treadmill walking sessions (OW-TW)**						
	**Stride duration variability**				**Gait parameters**		
	**Temporal organization**		**Regularity**	**Magnitude**	**Gait speed**	**Gait cadence**	**Step length**
	**H**	**α**	**SamplEn**	**CV**			
**CLINICAL PARAMETERS**							
Age	−0.28	−0.08	−0.11	0.18	−0.05	−0.06	−0.18
Disease duration	0.38	0.43	0.13	0.04	−0.29	0.16	−0.39
H&Y scale	0.60^**^	0.75^***^	−0.18	0.09	−0.35	0.20	−0.39
MDS-UPDRS III	0.56^*^	0.58^**^	−0.27	0.09	−0.32	0.22	−0.52^*^
BESTest	−0.51^*^	−0.64^**^	0.20	−0.19	0.55^*^	−0.11	0.73^***^
ABC-Scale	−0.22	−0.52^*^	−0.15	−0.20	0.63^**^	−0.13	0.66^**^
Gait speed change	0.14	−0.35	−0.05	−0.51^*^	**–**	0.42	0.96^***^

*The change expresses the difference between the two walking conditions (OW and TW)*.

*** p ≤ 0.001;

** p ≤ 0.01;

* *p ≤ 0.05. Bold data indicates statistically significant correlations (p < 0.05)*.

## Discussion

The aim of the present study was to investigate the immediate influence of treadmill walking compared to overground ambulation on both gait variability and spatiotemporal gait variables in Parkinson's disease and healthy controls. Firstly, our study demonstrated a more regular and temporally organized gait pattern in PD participants while healthy controls remained insensitive. A second observation was the highlighting of a reduced gait speed and a concomitant decreased step length, suggesting a cautious gait pattern adopted by PD participants walking on a treadmill. Finally, greater influence of TW was demonstrated among instable patients.

Complexity and regularity of stride time signals may reveal insight into the neurophysiological organization of locomotion and into the regulation of the interacting subsystems, constituting the locomotor system (Delignières and Marmelat, 2012; Hollman et al., 2016). Interestingly, the PD gait pattern was more temporally organized on treadmill, indicating strong coordination among the multiple sub-systems (Stergiou and Decker, 2011; Delignières and Marmelat, 2012). LRA exponents indeed yielded values close to 1 in PD, and were significantly higher than healthy age-matched controls where the temporal organization of gait variability was insensitive to TW (Table 2). Also, a more constrained and predictable locomotion is induced (i.e., reduction in SampEn) in PD patients walking on a treadmill while the healthy gait pattern remained indifferent to TW (Table 2). Therefore, although less complex gait dynamics would emerge from such constrained system, constraints induced by the treadmill belt could afford the necessary framework to regulate gait rhythmicity in PD while allowing some adaptations and not a completely rigid gait control.

Although some similarities are shared between overground and treadmill ambulation, TW implies specific physical and environmental constraints that are not present for OW (Hollman et al., 2016). Gait regulation could be modulated by physical constraints that reduce the number of available degrees of freedom. The intrinsic dynamics emerge from the multiplicative interactions that constitute the entire system including the locomotor system, the environment and their mutual history (Diniz et al., 2011). The coupling of the participant and environment modulates gait dynamics. Motor behavior emerges to satisfy the intrinsic and extrinsic constraints of the task at hand. Additionally, the strength of the coupling determines the quality of coordination within the organism–environment system (Diniz et al., 2011). The greater complexity and regularity of PD gait pattern on TW could be thus explained by a greater dependence on environmental conditions.

Mechanisms potentially involved in the therapeutic effect of the treadmill were identified by Bello and Fernandez-Del-Olmo who reviewed 10 years of investigation of treadmill training in PD (Bello and Fernandez-Del-Olmo, 2012). Although the mechanisms implicated in the treadmill effect on PD gait pattern remain elusive, PD patients could take advantage of the hip extension, the constant gait speed or the absence of visual flow on the treadmill (Bello and Fernandez-Del-Olmo, 2012). However, based on our experimental protocol, some mechanisms such as hand support or motor learning did not seem to explain the treadmill effect on PD gait pattern.

Adequate sensory inputs from TW may stimulate the spinal locomotor circuitry (i.e., Central Patterns Generators; CPG). Descendent supraspinal signals and afferent input of the limbs are suggested to regulate the CPG activity. In this way, lower limbs that are rhythmically moved backwards and behind the trunk can constitute a regulatory input from the environment that could restore some interaction within the locomotor system. Efficient, stable, and adaptable locomotor pattern requires complex dynamic sensorimotor interactions depending on highly complex processes including efferent-type (from the motor cortex, the cerebellum, the basal ganglia and central pattern generator) and afferent-type information coming from different peripheral feedbacks.

The use of regulatory inputs from the environment appears crucial to overcome the defective internal rhythm of the basal ganglia disorders (van Wegen et al., 2006; Baker et al., 2007; Nieuwboer et al., 2007). Greater propensity to be entrained by regulatory inputs characterizes PD gait control in comparison to healthy adults. Sensory inputs from TW could provide the necessary trigger to maintain some gait rhythmicity in the same way as external cues. From various nature (auditory, visual or proprioceptive and tactile), their usefulness in the struggle against the impaired gait automaticity was demonstrated in PD by bypassing the defective pallidocortical circuit (van Wegen et al., 2006; Chien et al., 2017; Dotov et al., 2017). In this way, TW could act as an external pacemaker and the fixed timing of leg movements could reduce the regulatory load on defective cortical areas allowing relative adaptations of gait parameters (Frenkel-Toledo et al., 2005; Snijders et al., 2011). Such adaptation was indeed highlighted on a 0.5-m-wide and 1.5-m-long treadmill (Bollens et al., 2014).

Similarly, the constant gait speed could also modulate the free regulation of walking in PD (Terrier and Dériaz, 2011) to such an extent that some authors postulated that the treadmill could function like a metronome (Malatesta et al., 2003). Although isochronic auditory stimulations are indeed suggested to afford the necessary framework to maintain gait rhythmicity in PD, evidence agrees that isochronic metronome breaks the complexity of both PD and healthy gait patterns (Dotov et al., 2017). Our results demonstrated TW influence only for PD population. Across existing literature, the influence of TW on the intrinsic gait dynamics appears indeed more equivocal for healthy population (Chang et al., 2009; Bollens et al., 2010; Terrier and Dériaz, 2011). The gait speed determination could be the key explicative factor. In our study, comfortable speed individually determined was preferred to an imposed speed applied for all subjects. Influence of TW in healthy population was indeed highlighted in studies where the gait speed was not individually determined which could have induced higher constraints on gait control (Terrier and Dériaz, 2011). Speed determination is usually questionable in studies conducted on LRA in many domains (Gilden et al., 1995; Hausdorff, 2009; Wijnants et al., 2012). In addition to the duration of walking sessions and the use of handrails, the gait speed determination ranks among the methodological considerations that could explain differences from a similar study conducted earlier by Frenkel-Toledo et al. Handrails use artificially improves gait stability and walking sessions lasted 2 min while gait variability approaches are usually data-hungry techniques. Also, gait speed imposed on treadmill in the study of Frenkel-Toledo et al. corresponded to the gait speed of the walking session with a walker which is known to be not representative of the spontaneous gait pattern (Kegelmeyer et al., 2013).

The damaged automaticity in PD gait pattern can also be overcome by the use of attentional strategies (Baker et al., 2007; Warlop et al., 2017). Among the environmental constraints of TW, the absence of optical flow could reduce distraction and allow the patients to allocate attentional resources on the task at hand (Bello and Fernandez-Del-Olmo, 2012). Attentional allocation could be highlighted by the higher magnitude of the stride duration variability observed in PD participants, which could be particularly increased given the newness of the task and the fear of falling expressed by some patients. However, the gait speed seems to be a better candidate to explain such an increase (Table 5). Walking on a treadmill does not seem to require greater involvement of attentional resources in both groups since the magnitude of gait variability became not significantly higher on TW than OW when parameters were normalized for gait speed (Table 4). Also, a previous study highlighted that PD gait pattern seems to be more dependent on sensory inputs generated by the belt movement than on attentional resources (Bello et al., 2010). Thus, in accordance with the existing literature, this study extends previous observations that gait speed is tightly linked to the magnitude of gait variability (Bollens et al., 2012).

Gait pattern modifications could also be induced by the newness of task despite a familiarization period. Treadmill walking modulated classical spatiotemporal gait parameters in PD population. Compared to overground ambulation, gait speed and step length were clearly decreased in PD participants walking on treadmill while only slight changes were observed in healthy controls. In parallel, cadence of PD gait pattern increased, acting as a compensatory mechanism. Reduced gait speed and step length ranks among other gait parameters modifications that characterize precautious gait pattern highlighted in frailty patients (Herman et al., 2005; Balash et al., 2007). Indeed, PD patients presented lower balance performance and higher level of fear of falling than healthy age-matched controls (Table 1).

Gait pattern seems indeed to be the most influenced by TW in patients with the most precarious balance status (Figure 1, Table 5). The temporal organization of gait variability was recently suggested as a gait stability index (Hausdorff, 2009; Warlop et al., 2016). Indeed, the more random the walking pattern (i.e., Hurst and α exponents close to 0.5 and to 0, respectively), the greater influence of TW is observed. The most important improvement of gait dynamics was thus observed in patients with the greater instability. As largely reported in literature (Herman et al., 2005; Balash et al., 2007), balance status also greatly explains the cautious gait pattern adopted by the most instable patients.

Importantly, all of those modifications observed in PD population walking on a treadmill arise the question of adequate gait assessment including gait variability on TW in PD participants. This study highlights the necessity to cautiously consider the use of motorized-treadmill in gait variability assessment as well as classical spatio-temporal gait parameters in gait analysis and usual clinical practice. Hence, gait disorders resulting from the insufficiency of internal rhythm generation is the most accurately depicted on OW given the higher propensity of PD population to be influenced by external rhythmical inputs. Also, treadmill walking holds a special place in Parkinson's disease (PD) as an efficient and promising rehabilitative approach to improve classical spatiotemporal gait disorders even over the long-term (Mehrholz et al., 2015). While little is know about the treadmill walking influence on LRA, this study highlights its potential of restoration or modulation of the temporal organization of gait variability in PD. Similarly to studies of forced use and exercise conducted on animal models, continuous treadmill belt's rolling out imposes to continuously walk by offering to PD patients the necessary external cues that may be also induced neurochemical changes over time (Tillerson et al., 2001).

The present study has some limitations that should be addressed. A safety harness was used to ensure the patient's security while some studies have highlighted influences of TW on vertical trunk acceleration and lower limb kinematics (Aaslund and Moe-Nilssen, 2008; Decker et al., 2012). Influences are particularly evident with body weight support and even more with static body weight support (Aaslund and Moe-Nilssen, 2008; Kyvelidou et al., 2008). However, the body weight support was set at 0% in our study since, when interval variables are the primary interest, the emergence of natural gait dynamics did not seem to be disrupted by the non-weight support harness (Stout et al., 2016). Similarly to backpack or hydrotherapy treatments, the safety harness could also provide the necessary sensory trick to struggle against postural instability in PD (Gerton et al., 2010; Vivas et al., 2011). Familiarization period can also constitute a limitation of the present study. Actually, no guidelines were edited with respect to the duration of the familiarization period (Wass et al., 2005; Van de Putte et al., 2006; Schellenbach et al., 2010). A familiarization period of 5 min was determined based on a previous study conducted on stroke patients to allow adaptation to TW and avoid any fatigue (Bayat et al., 2005). As suggested by our observations, PD gait dynamics could be modulated by the rhythmical inputs generated by the motorized treadmill. Therefore, it could be interesting to investigate the influence of non-motorized treadmill to better elucidate the participation of the motor-driven part of the TW compared to the common parts shared with the overground ambulation.

In conclusion, the present study highlights that the treadmill could afford the necessary framework to regulate gait rhythmicity in PD while precautious gait pattern seems to be adopted with such type of walking. A more temporally organized and more regular gait pattern emerges from treadmill walking in PD while no influence was observed in healthy gait pattern. Those observations support the hypothesis of a greater dependence to regulatory inputs as an explanatory factor of treadmill influence observed in PD. Our results may form a further step toward a better understanding of the effect of treadmill on PD gait. Also, the present findings underline that gait analysis using treadmill devices should be cautiously considered in PD and especially for gait variability assessment.

## Author contributions

TW assisted with participant recruitment and data collection; participated in data analyses and interpretation of results; did literature searches; and drafted and wrote the manuscript. CD provided methodological and statistical inputs to the study, largely contributed to the good application of reference mathematical methods; participated in the interpretation of results; helped to draft the manuscript; and revised the manuscript. GS and TL provided methodological input to the study; participated in the interpretation of results; and revised the manuscript. AJ was the chief investigator who oversaw and coordinated all study activities; assessed participant eligibility; provided advice and input to all methodological issues; participated in the interpretation of results; helped to draft the manuscript; and revised the manuscript. All authors read and approved the final manuscript.

### Conflict of interest statement

The authors declare that the research was conducted in the absence of any commercial or financial relationships that could be construed as a potential conflict of interest.
